# Impact of a Nanoscale Iron–Chlorobenzene Mixture on Pulmonary Injury in Rat Pups: Extending Exposure Knowledge Using Network Technology

**DOI:** 10.3390/toxics13030221

**Published:** 2025-03-17

**Authors:** Kezhou Liu, Ying Xu, Mengjie Ying, Meiling Chen

**Affiliations:** 1 School of Automation (Artificial Intelligence), Hangzhou Dianzi University, Hangzhou 310018, China; 221060398@hdu.edu.cn (Y.X.); jsl_coder@163.com (M.Y.); 2 School of Environment and Resources, Zhejiang University, Hangzhou 310058, China; 12114009@zju.edu.cn

**Keywords:** network toxicology, chlorobenzene, photochemical conversion, pulmonary development, oxidative stress, inflammatory factors

## Abstract

Particulate matter coexists with persistent organic pollutants (POPs) in the atmosphere, which can enter the human body by accompanying inhalable particles in the respiratory tract. Photochemical conversion further alters the chemical composition of the precursor particles and secondary products. This study investigated the effects of nanoscale iron–chlorobenzene mixtures and their photochemical conversion products on early lung development in rat pups. Using network toxicology and animal experiments, we constructed a compound toxicity–target network and developed air exposure models. This study revealed that both pollutants, before and after photochemical conversion, bound to the aryl hydrocarbon receptor (AhR), increased oxidative stress, altered lung tissue morphology, and reduce inflammatory factor expression. Rat pups were highly sensitive to pollutants during critical stages of lung development. However, no significant differences in oxidative stress or inflammation were observed between the pollutants, likely because of immature lung tissues. Once tissue damage reached a threshold, the response to increasing pollutant concentrations diminished. This study provides insights into atmospheric pollutant toxicity and scientific evidence for the risk assessment of dioxin-like nanoscale mixtures.

## 1. Introduction

Persistent organic pollutants (POPs) are a significant component of organic matter in atmospheric particulate matter. These compounds are characterized by their persistence, resistance to degradation, bioaccumulation, long-range transport, and high toxicity. Among POPs, dioxins differ from traditional natural toxins in that they are exclusively anthropogenic in origin, resulting from by-products of combustion processes and various industrial activities [1,2]. These organic pollutants have low boiling points and typically exist in the gaseous phase at ambient temperatures. Furthermore, they readily adsorb onto fine particles in the environment, thereby facilitating their dispersion in the atmosphere [3].

In China, the steel industry is a significant emission source of polychlorinated dibenzo-p-dioxins and dibenzofurans (PCDD/Fs) [4]. Studies have shown that during the sintering process of the steel industry, precursor substances such as chlorophenols, chlorobenzenes, and polychlorinated biphenyls (PCBs) can form in the flue gas through low-temperature reactions and further undergo catalytic chlorination reactions on the surface of solid fly ash to generate PCDD and PCDF [5,6]. Notably, China’s production and emission of chlorobenzenes (CBs) account for more than 60% of the global total [7]. This further exacerbates the risk of PCDD/F formation in the environment. Moreover, photochemical conversion is considered to be an important pathway for the formation of PCDD/Fs and plays a crucial role in the complex process of generating secondary products from high-emission precursors [8,9]. Under photochemical conversion conditions in an atmospheric environment, adsorbed dioxin compounds may undergo physical or chemical reactions with other gaseous pollutants, leading to the formation of secondary particulate matter. This process not only increases the complexity of particulate matter but also may alter its toxicity profile [10,11].

These modified particles can be inhaled into the lungs through respiration, potentially causing toxic effects on the respiratory system [12]. Notably, children and adolescents, whose lung tissues are more sensitive during growth and development, are at greater risk of these toxic effects [13,14]. At birth, the alveoli of newborns are not fully mature, and the alveolarization process continues until approximately the age of 3 years. In rodent models, the lungs of neonatal rats are in the saccular stage of development, with the alveolar stage beginning at approximately postnatal day 5 [15,16]. Therefore, establishing a neonatal rat exposure model is important for studying the damage mechanisms of particulate pollutants in the early stages of lung development. Moreover, exposure to environmental pollutants is significantly associated with the development of lung function, respiratory infections, and asthma in children. These toxic effects pose greater risks, potentially disrupting normal lung function and leading to long-term health issues [17,18,19].

In this study, we utilized network toxicology approaches to identify the primary mechanisms by which dioxin-like compounds cause lung injury. Compared with traditional toxicological testing methods that focus on one or a few targets, network toxicology allows for a faster and more comprehensive assessment of the complex biological effects of potential pollutants [20]. By leveraging extensive toxicological data on 2,3,7,8-tetrachlorodibenzo-p-dioxin (TCDD) [21] and applying network toxicology methodologies, we conducted an in-depth identification and analysis of the toxicity pathways involved in TCDD-induced lung injury. This approach helped to elucidate the toxicological characteristics of TCDD, predict its potential toxicity and molecular mechanisms, and extend these findings to other dioxin-like compounds.

In this study, we selected iron(III) oxide (α-Fe_2_O_3_) nanoparticles and chlorobenzene (CB), a persistent organic pollutant, as precursors for the reaction. Two types of pollutants with different toxicities were generated with and without photochemical conversion. In this experiment, α-Fe_2_O_3_ primarily served to promote the formation of secondary products [22]. Air exposure was used to assess the degree of lung injury caused by these pollutants in neonatal rats during the early developmental stages. This study provides new insights into effective and rapid strategies for evaluating the toxicity of environmental pollutants and lays a foundation for diagnosing diseases associated with exposure to these toxic substances.

## 2. Materials and Methods

### 2.1. Preparation of Pollutants

The pollutants in this study were independently prepared by our research group [23]. The primary materials used were as follows: chlorobenzene CB (500 ppmv under a N_2_ atmosphere), purchased from Jingong Special Gases Co., Ltd., Hangzhou, China; iron(III) oxide (α-Fe_2_O_3_, purity of 99.8%, pore size of ca. 30 nm, and surface area of 137.8 m^2^/g), purchased from Aladdin Biochemical Technology Co., Ltd., Shanghai, China; anhydrous ethanol, purchased from China National Pharmaceutical Group Corporation (Sinopharm), Beijing, China; and a xenon lamp (wavelength range of 320–780 nm; model PLS-SXE300), purchased from Pofeilai Technology Co., Ltd., Beijing, China.

The preparation steps were as follows: First, anhydrous ethanol was used to clean and moisten the circular sample. Then, 0.15 g of α-Fe_2_O_3_ powder was evenly applied to the surface. After the ethanol evaporated naturally, the sample stage was placed in the reactor. The reactor was a double-layer sealed container equipped with a recirculating water system and an internal magnetic stirrer and was wrapped with fiberglass on the outside for insulation and to prevent exposure to light. The temperature of the outer chamber of the reactor was maintained at 30 °C by using a recirculating water system.

Next, a portable syringe was used to introduce 200 ppm (CB) into the reactor chamber. The mixture was stirred for ~20 min to ensure homogeneous mixing. A xenon lamp was subsequently turned on for 7 h to induce photochemical reactions. This process was repeated five times. Alternatively, the xenon lamp was left off and the reaction was conducted in the dark for 7 h, which was repeated five times. Under both conditions, the reactions were conducted for a total of 35 h to prepare pollutant samples before and after photochemical induction.

### 2.2. Experimental Animals

Eleven SPF-grade SD rat pups aged one day were purchased from the Zhejiang Academy of Animal Medical Sciences. The pups were housed under controlled conditions at a temperature of 24 ± 2 °C and a relative humidity of 50–70%. They were nursed by their dams, which were provided free access to food and water.

### 2.3. Network Toxicology Prediction

The data required for this study were obtained from online database platforms.

#### 2.3.1. Identification of TCDD Target Proteins

Certain congeners of dioxin, furan, and PCB families are referred to as dioxin-like compounds because their chemical structure, physicochemical properties, and toxicological responses are similar to those of the indicator chemical TCDD [24].

The standard structure and SMILES string of TCDD were determined from a search of the PubChem database. With “TCDD” used as the keyword, the potential targets of TCDD were retrieved from the ChEMBL database, with the species restricted to *Homo sapiens*. The SMILES string of TCDD was subsequently uploaded to the SWISS database to identify overlooked targets. The ChEMBL and SWISS IDs of the potential targets were integrated and duplicated. The names of the obtained targets were standardized via the UniProt database. Finally, the combined targets were used to construct a TCDD target library.

#### 2.3.2. Identification of Intersecting Genes Between TCDD and Lung Injury

Relevant target information associated with lung injury was retrieved from the GeneCards and OMIM databases using “lung injury” as the keyword. A Venn diagram was used to identify the common potential targets between TCDD and lung injury. These intersecting genes are considered to be potential targets for TCDD-induced lung injury.

#### 2.3.3. Construction of Protein–Protein Interaction Network

The intersecting genes representing potential targets for TCDD-induced lung injury were input into the STRING database, with the species restricted to *Homo sapiens*. The results generated via STRING were then imported into Cytoscape software (version 3.10.2), a network biology visualization and analysis tool, to construct a protein–protein interaction (PPI) network.

#### 2.3.4. Enrichment Analysis of Intersection Genes

Information on potential targets for TCDD-induced lung injury was input into the Metascape database for the Gene Ontology (GO) enrichment analysis and Kyoto Encyclopedia of Genes and Genomes (KEGG) enrichment analysis.

### 2.4. Animal Experimental Validation

#### 2.4.1. Animal Grouping and Establishment of the Air Exposure Model

1. Animal Grouping

**Figure 1:**
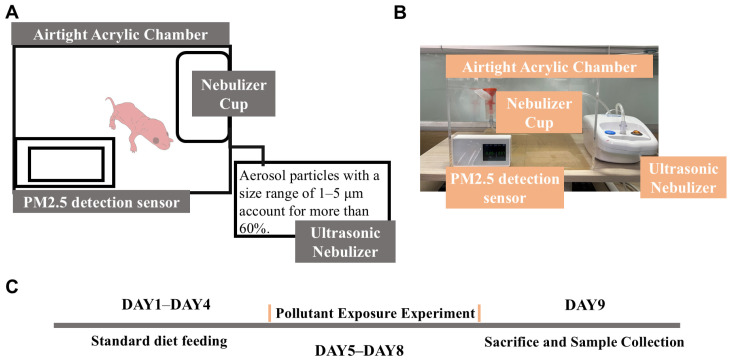
Air exposure experimental setup and procedure schematic. (**A**) Schematic diagram of setup. (**B**) Photograph of setup. (**C**) Experimental procedure flowchart.

On postnatal day 1, SD rat pups were nursed by their dams for four days and then randomly divided into the following three groups: a group without photocatalysis exposure (*n* = 4), a group with photocatalysis exposure (*n* = 4), and a control group (*n* = 3). Pollutant air exposure was conducted from postnatal days 5 to 8. The animals were sacrificed by cervical dislocation 24 h after the final exposure, and tissues were collected for analysis (Figure 1C).

2. Air Exposure Model

An air exposure system was constructed to simulate pollutant exposure in a controlled environment. The system consisted of three main components.

- Construction of the exposure chamber: a large transparent acrylic board was used to construct the main chamber.
- Generation of pollutant aerosols: a compressed nebulizer (NB-212C; Ludei Medical Devices, Shanghai, China) was employed to generate aerosols containing pollutants mixed with pure water, which were then introduced into the exposure chamber.
- Monitoring of the PM2.5 concentration: a PM2.5 sensor was used to continuously monitor the concentration of PM2.5 inside the sealed chamber to ensure consistent exposure conditions (Figure 1A).

To establish the air exposure model, suspensions of pollutants were prepared by mixing the pollutants before and after photocatalysis with pure water to create suspensions at a concentration of 1 mg/L. The automatic nebulization system was activated, and the air compressor delivered compressed air into a plastic tube containing 1 mg/L of the pollutant solution. The pollutants were aerosolized and introduced into a sealed chamber housing the rat pups, where they were exposed for 1 h. During this period, the PM2.5 concentration inside the chamber was monitored via a PM2.5 detector (Figure 1B).

#### 2.4.2. Histopathological Examination

After the rat pups were subjected to cervical dislocation, the right upper lobe of the lung was immediately excised and immersed in 4% paraformaldehyde for 24 h. The fixed tissues were then sent to the Public Technology Platform of Zhejiang University School of Medicine for hematoxylin and eosin (H&E) staining. The tissue sections were observed via an inverted fluorescence microscope (Olympus, Tokyo, Japan) to examine the morphological changes in the tissues.

#### 2.4.3. Measurement of Oxidative-Stress-Related Markers in Tissue

Fresh lung tissues were collected and homogenized to prepare a 10% (*w*/*v*) lung tissue homogenate by adding 90 µL of PBS to 10 mg of tissue. The homogenate was centrifuged at 12,000 ×*g* for 3 min at 4 °C via a refrigerated centrifuge (Thermo Fisher, Waltham, MA, USA). The supernatant was collected, and a portion of the 10% tissue homogenate was used to measure the protein concentration via a BCA assay kit (Beyotime, Shanghai, China). Additionally, the activity and content of superoxide dismutase (SOD) and glutathione (GSH) in the lung tissues were determined according to the manufacturer’s instructions for the respective assay kits (Beyotime, Shanghai, China).

#### 2.4.4. Determination of Inflammatory Cytokine RNA Expression in Tissue

Twenty milligrams of lung tissue was placed into a 1.5 mL RNase-free centrifuge tube and rapidly homogenized with 600 µL of an ice-cold lysis buffer via a tissue grinder. Total RNA was extracted from the homogenates according to the instructions of the animal RNA extraction kit (Beyotime, Shanghai, China). The concentration and purity of the RNA samples were measured via a nanophotometer (Thermo Fisher, Waltham, MA, USA). The qRT–PCR mixture was prepared according to the instructions of the qRT–PCR premix kit (Beyotime, Shanghai, China), and the target and internal reference genes were loaded in triplicate. The reaction mixture was centrifuged at 500 r/min for 30 s and transferred to a 96-well PCR plate. Real-time PCR was performed via a Roche LightCycler 480 system (Roche, Basel, Switzerland) with the following cycling conditions: 50 °C for 30 min (reverse transcription) and 95 °C for 2 min (initial denaturation), followed by 40 cycles of 95 °C for 15 s (denaturation) and 60 °C for 30 s (annealing and extension). The relative expression levels of the target genes were normalized to those of β-actin and calculated via the 2^−ΔΔCT^ method. The PCR primers used were synthesized by Sangon Biotech (Shanghai, China) (Table 1).

#### 2.4.5. Statistical Analysis

The data were presented as the means ± standard deviations (means ± SDs), and were visualized via graphs generated via GraphPad Prism 9. Comparisons between groups were performed via the independent sample *t*-test. Statistical significance was set at 0.05.

## 3. Results

### 3.1. Validation Results of Network Toxicology

#### 3.1.1. Prediction of TCDD-Induced Lung Injury Targets

**Figure 2:**
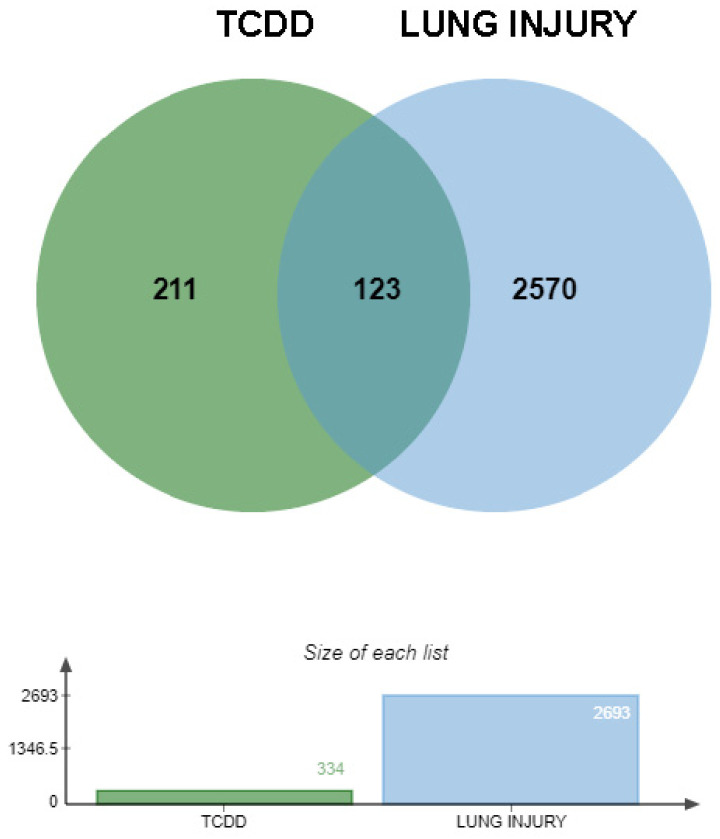
Venn diagram of TCDD targets and lung injury targets.

In this study, we initially identified 344 potential targets of TCDD via the ChEMBL and SWISS databases. Additionally, 2693 targets highly associated with lung injury were retrieved from the GeneCards and OMIM databases. By integrating and deduplicating these target sets, 123 intersecting targets were obtained, which were considered to be potential targets for TCDD-induced lung injury (Figure 2).

A Venn diagram was used to illustrate the overlap between the targets and those associated with lung injury. The 344 TCDD targets identified from the ChEMBL and SWISS databases were intersected with the 2693 lung injury-related targets determined through the analysis of the GeneCards and OMIM databases. Notably, the overlapping regions between these two datasets revealed 123 potential targets specifically related to TCDD-induced lung injury.

#### 3.1.2. Interaction Network of Potential Targets

**Figure 3:**
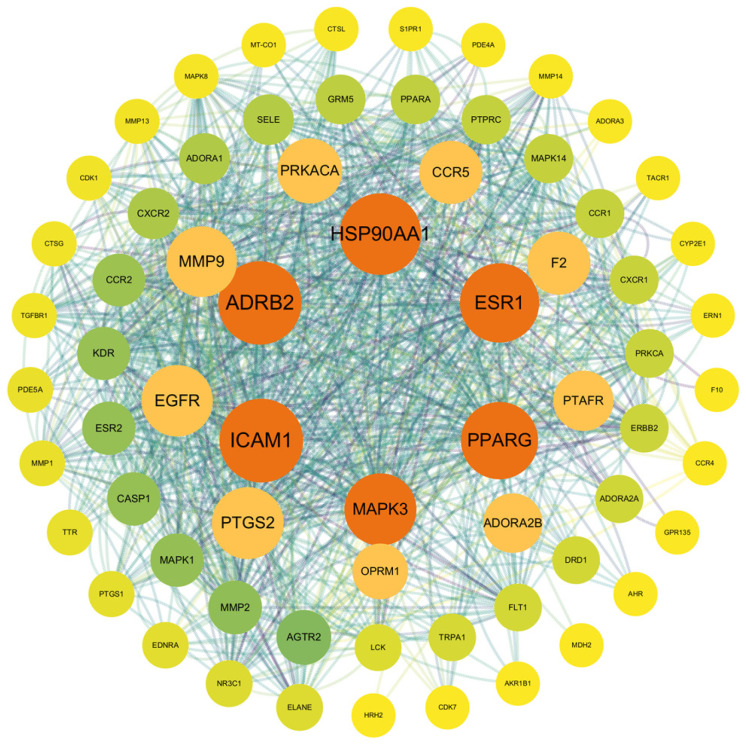
PPI network diagram of the intersection targets between TCDD and lung injury.

A protein–protein interaction (PPI) network was constructed via the STRING database, resulting in a network with 67 nodes and 469 edges, with an average node degree of 14. The topological properties of the network nodes were analyzed via Cytoscape software. On the basis of the betweenness centrality (BC) calculated via the CytoNCA plugin, the following six core targets were identified: ICAM1, ADRB2, HSP90AA1, ESR1, PPARG, and MAPK3. A visualized and optimized PPI network was generated (Figure 3). In this network, larger nodes indicated greater representativeness and importance. The size and color intensity of the nodes were proportional to their degree values, with larger and darker nodes representing higher connectivity.

The top three core proteins identified were ICAM1, ADRB2, and HSP90AA1. These proteins are widely recognized for their critical roles in various cellular functions, including the regulation of intracellular cAMP levels, which controls multiple physiological processes, protects against cellular stress, and modulates inflammatory and immune responses.

Core proteins, such as ICAM1, ADRB2, HSP90AA1, ESR1, PPARG, and MAPK3, play essential roles in cellular functions. Notably, HSP90AA1 is crucial for the cellular stress response. Exposure to TCDD may disrupt the function of these proteins, thereby exacerbating oxidative stress, a key factor in the development of lung injury and disease [25,26].

#### 3.1.3. Enrichment Analysis of Intersecting Targets

**Figure 4:**
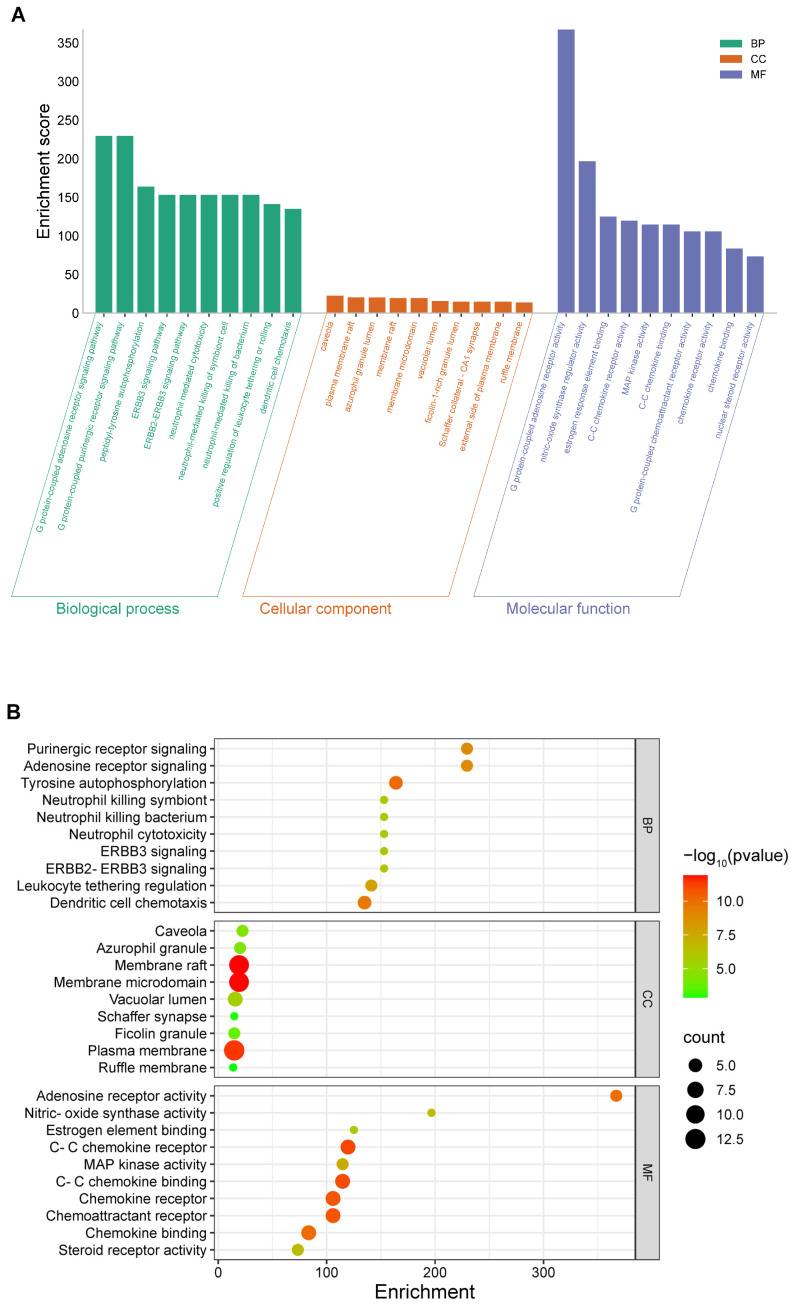
GO functional enrichment (top 10). (**A**) Significant enrichment patterns of 123 potential targets from the GO enrichment analysis. This panel displays the significant enrichment patterns for the top 10 enriched terms among 123 potential target genes in the GO enrichment analysis. The figure emphasizes GO categories with lower FDR values, including biological BP, CC, and MF. (**B**) Visualization of the results of the GO enrichment analysis: the results are presented via a bubble chart in which the size of each bubble correlates with the gene expression level within a specific pathway. The intensity of the bubble color visually indicates the significance of the enrichment, with deeper colors signifying more significant enrichment.

We performed a GO analysis on the 123 potential targets via the Metascape database, with the species restricted to *Homo sapiens*. Our analysis revealed 984 statistically significant GO terms comprising 842 biological process (BP), 58 cellular component (CC), and 84 molecular function (MF) terms. GO terms were ranked on the basis of false discovery rate (FDR) values. The top ten terms with the lowest FDR values in each category (BP, CC, and MF) were selected and visualized in the enrichment analysis plot (Figure 4).

This approach allowed us to identify key biological processes, cellular components, and molecular functions that were significantly associated with potential targets of TCDD-induced lung injury. The Metascape database facilitated a comprehensive and systematic analysis of these targets, providing valuable insights into the underlying mechanisms of TCDD-induced lung injury.

**Figure 5:**
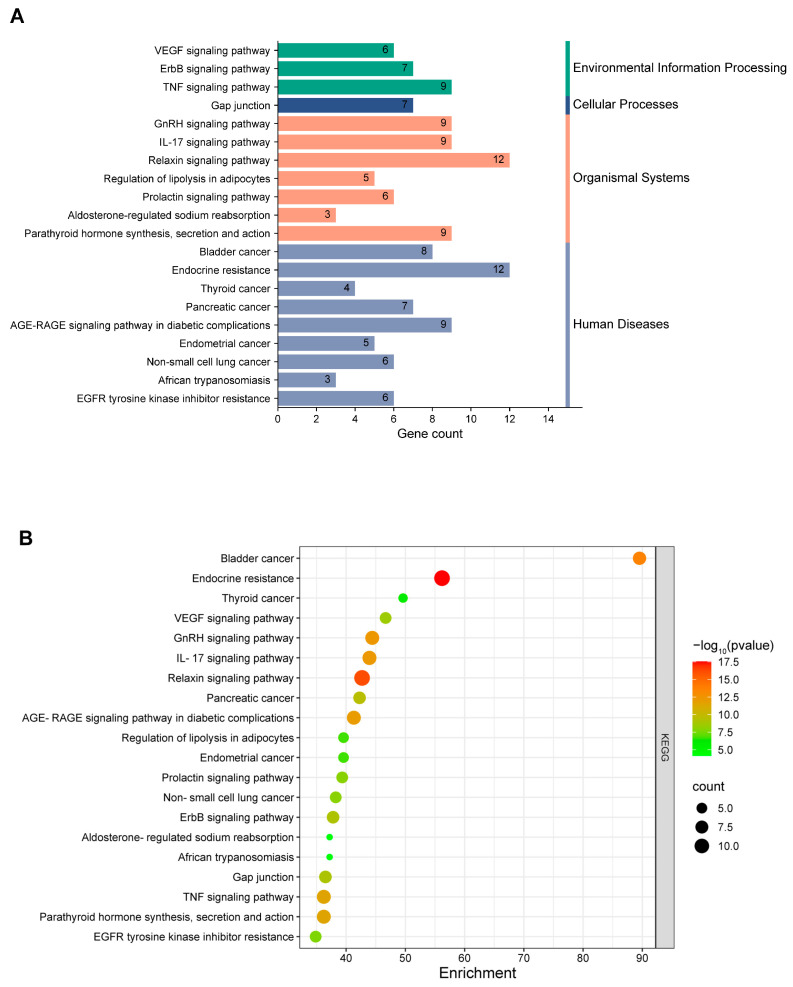
KEGG pathway enrichment analysis. (**A**) Histogram of enrichment frequencies and statistical significance. The histogram clearly displays the enrichment frequency and statistical significance of each pathway. This study provides a concise and intuitive description of the top 20 most significantly enriched KEGG signaling pathways. (**B**). Bubble chart of the top 20 enriched KEGG pathways. The bubble chart, arranged in descending order of FDR values, shows the top 20 significantly enriched KEGG signaling pathways.

We performed a KEGG pathway enrichment analysis on the 123 potential targets via the Metascape database to identify the specific signaling pathways in which they were involved. A total of 155 pathways were enriched, and we generated a significant bubble plot and categorized histogram to visually represent the top 20 KEGG pathways on the basis of the inverse order of their FDR values (Figure 5).

#### 3.1.4. Functional Insights from GO and KEGG Enrichment Analyses

GO and KEGG enrichment analyses of the potential targets revealed that these genes were involved in several critical regulatory processes that contribute to lung injury. The GO enrichment analysis revealed that the genes were associated with cell growth and differentiation, neutrophil cytotoxicity, and cell signaling and function, which can lead to abnormal cellular responses in the lungs. The primary pathways identified in the KEGG enrichment analysis included inflammation, cell proliferation and survival, intercellular communication, metabolic regulation, and signal transduction.

The PPI analysis, along with the KEGG pathway and GO enrichment analyses, revealed that activation of AhR by TCDD [27,28] led to phosphorylation and activation of EGFR. As a key node, EGFR interacts with various proteins and signaling pathways, playing a crucial role in activating downstream signaling pathways such as the MAPK and PI3K pathways [29]. The MAPK pathway may promote the expression of genes related to oxidative stress responses, whereas the PI3K/AKT pathway is central to cell survival and antioxidant stress [30,31]. AKT activation can increase the expression of antioxidant enzymes such as superoxide dismutase (SOD), thereby reducing the accumulation of reactive oxygen species (ROS) [32]. Conversely, inhibition of the PI3K/AKT pathway may lead to ROS accumulation and exacerbate oxidative stress [33].

Additionally, our analysis revealed the enrichment of inflammation-related signaling pathways such as the tumor necrosis factor (TNF) and interleukin-17 (IL-17) signaling pathways. Abnormal activation of these pathways may indicate the dysregulation of toll-like receptor (TLR) responses, leading to aberrant inflammatory reactions. When TLRs bind to their ligands, they activate the NF-κB pathway, which is downstream of TLR signaling [34,35,36]. NF-κB regulates immune responses and inflammation through the transcriptional regulation and activation of cytokines and chemokines. AhR signaling is associated with dysregulated TLR responses, and studies have shown that crosstalk between AhR and NF-κB [37,38] results in mutual inhibition of the two pathways. The simultaneous activation of NF-κB and AhR may lead to a dysregulation of cytokine transcription, such as increased interleukin-1β (IL-1β) and interleukin-8 (IL-8) and decreased interleukin-12α (IL-12α) and interleukin-6 (IL-6) [39].

### 3.2. Animal Experiment Results

#### 3.2.1. Characterization of Pollutants Before and After Light Induction

**Figure 6:**
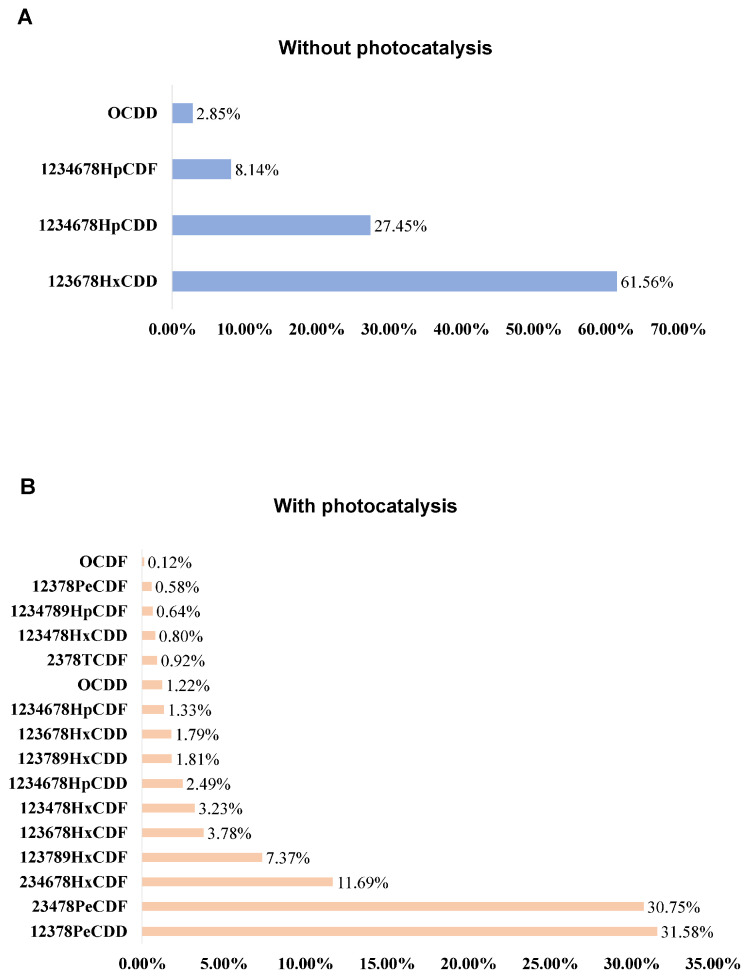
Dioxin-like substance content proportions. (**A**) The percentage of dioxin-like substances in the absence of photocatalysis and (**B**) the percentage of dioxin-like substances in the presence of photocatalysis.

After a 35 h reaction in a dark environment, the primary secondary products detected on the surface included four compounds, notably, 1,2,3,6,7,8-HxCDD and 1,2,3,4,6,7,8-HpCDD. In contrast, following a 35 h reaction under light exposure, a total of 16 secondary products were detected, with major compounds including 1,2,3,7,8-PeCDD and 2,3,4,7,8-PeCDF. The specific compositions and relative proportions of these compounds are shown in Figure 6.

The analysis of the indoor reaction by-products confirmed that CB could be converted into dioxins under both light and dark conditions. Under light conditions, CB undergoes dechlorination and nucleophilic substitution on α-Fe_2_O_3_, leading to a significant increase in the amount of reaction by-products [40]. After photocatalysis, the number of secondary products significantly increased from four to sixteen. Dioxins, which have stable structures and long half-lives, can persist in the body after just one exposure, and prolonged exposure may result in their accumulation within the body.

#### 3.2.2. Histopathological Results of Lung Injury Caused by Pollutants with and Without Photocatalysis Exposure

**Figure 7:**
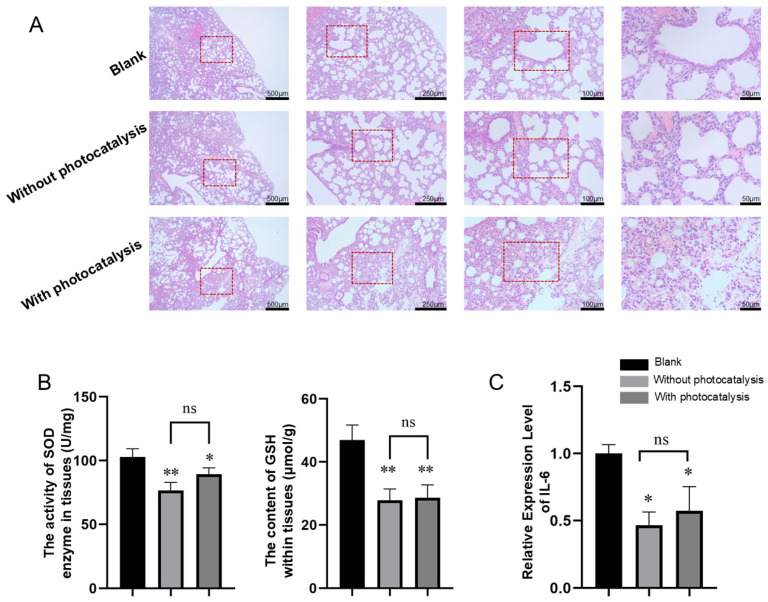
Animal experiment results: (**A**) lung histopathology of suckling rats with HE staining, (**B**) analysis of oxidative stress levels (* indicates *p* < 0.05, ** indicates *p* < 0.01, and ns indicates *p* > 0.05), and (**C**) expression of the inflammatory cytokine IL-6 (* indicates *p* < 0.05, and ns indicates *p* > 0.05).

To verify the pathological changes in the lung tissues of neonatal rats caused by pollutants with and without photocatalysis, we conducted lung sampling and pathological examinations of the rats in each group. As shown in Section 3 in Figure 7A, the lung tissue of the control group maintained a normal structure, with clear alveoli and normal septa, without significant cellular infiltration or fibrosis. Exposure to a mixture of gases without photocatalysis resulted in disorganized lung tissue, thickened alveolar septa, and significant cellular infiltration. Potential signs of fibrosis were also observed in the images. Exposure to a mixture of gases with photocatalysis caused more severe lung damage, with noticeable deformation of the alveolar structure, thickening of the alveolar walls, infiltration of inflammatory cells, and significant disruption of alveolar integrity, leading to severe structural damage to the lung tissue.

#### 3.2.3. Effects of Pollutants with and Without Photocatalysis on the Activity or Content of SOD and GSH in Lung Tissue

The results for the oxidative-stress-related biochemical indicators are shown in Figure 7B. Compared with those in the control group, the levels of SOD and GSH in the lung tissue of neonatal rats exposed to pollutants without photocatalysis were significantly lower (*p* < 0.01). In the lung tissue of neonatal rats exposed to photocatalytic pollutants, SOD levels were decreased (*p* < 0.05) and GSH levels were significantly reduced (*p* < 0.01). No significant differences were observed between the two groups.

#### 3.2.4. Effects of Pollutants with and Without Photocatalysis on the Expression of IL-6 in Lung Tissue

The relative expression of IL-6 in the tissues of neonatal rats measured by qRT–PCR is shown in Figure 7C. Compared with that of the control group, the relative expression of IL-6 in the lung tissue of neonatal rats exposed to pollutants without photocatalysis was significantly lower (*p* < 0.05). The relative expression of IL-6 in the lung tissue of neonatal rats exposed to photocatalytic pollutants was also significantly reduced (*p* < 0.05). No significant differences were observed between the two groups.

## 4. Discussion

The present study employed a combined approach of network toxicology and animal experiments to thoroughly investigate the molecular mechanisms underlying pulmonary injury induced by exposure to nanoscale iron–chlorobenzene mixtures with and without photocatalysis. Our findings revealed a dual mechanism characterized by oxidative stress dominance and an inflammatory pattern shift during the developmental stage of the lung.

This mechanism was consistent with previous studies on the role of oxidative stress and inflammation in lung injury. An increase in oxidative stress is related to the accumulation of ROS induced by pollutants. The oxidative stress biomarkers in the lung tissues of neonatal rats, as well as the levels of GSH and SOD, demonstrated that the oxidative stress levels in the lung tissues of neonatal rats exposed to pollutants for four days were significantly elevated, with concomitant structural lesions in the lungs. Although prolonged exposure to iron oxide particles can also increase oxidative stress levels in lung tissues, short-term low-dose exposure to iron oxide is considered insufficient to cause pulmonary injury according to existing studies [41,42]. However, long-term high-dose exposure to iron oxide particles leads to oxidative stress damage, which is often characterized by the aggregation of particles in the alveoli and a significant increase in the expression of the inflammatory cytokine IL-6 [43]. In our study, the short-term exposure paradigm was employed. The reduction in oxidative stress levels and inflammatory cytokine expression in the experimental group could be attributed to the distinct mechanisms by which the nanoscale iron–chlorobenzene mixture and the two dioxin-like pollutant mixtures (with and without photocatalysis) induced damage to the lung tissue. Sustained accumulation of ROS imposes stress on the intracellular antioxidant defense system [44]. In this context, antioxidants such as SOD and GSH, which are essential for neutralizing excessive ROS, are gradually depleted. As ROS levels increase, SOD activity may decrease due to overload and the GSH content may also decrease because of continuous antioxidant responses [45]. In the absence of timely ROS clearance, these ROS can cause cellular damage, leading to structural changes in lung tissue [46,47,48].

The network toxicology analysis further revealed that nanoscale iron–chlorobenzene mixtures primarily contributed to lung damage through oxidative stress, and the toxicological targets of dioxin compounds, including the dysregulation of HSP90AA1, MAPK3, and ADRB2, were central to oxidative damage. Dysfunction of HSP90AA1 impairs the normal function of the cellular antioxidant defense system [49]. The aberrant activation of MAPK3, on the other hand, promotes the excessive generation of ROS through multiple pathways [50], where the accumulation of ROS in the lung tissue remains unchecked, resulting in structural damage. Therefore, it can be concluded that oxidative stress and ROS accumulation play pivotal roles in lung injury caused by these mixtures. In exploring the further impact of inflammatory responses, we observed that exposure to mixtures led to a reduction in the expression of the inflammatory factor IL-6, suggesting that pollutants have an inhibitory effect on lung inflammation. IL-6, a key cytokine, plays a regulatory role in inflammation. A decrease in IL-6 expression implies a limited ability of the lungs to repair damage, thus exacerbating the severity of lung injury [51,52]. According to predictions from network toxicology, pollutants may interact with the inflammatory NF-κB pathway through the activation of AhR, thereby inhibiting IL-6 expression. Previous studies have shown that AhR activation significantly regulates inflammation and affects cytokine expression, which may be one of the mechanisms through which pollutants suppress IL-6 expression [53]. The pathway enrichment analysis revealed that, unlike the typical acute inflammatory response, the IL-17/TNF pathway preferentially activates inflammatory cytokines that are independent of IL-6 (such as IL-1β and TNF-α). In contrast, the neonatal rat model used in this study represented the stage of lung development. During this period, developing lung tissue has an inherent self-repair capacity. In response to oxidative-stress-induced damage, this repair ability may help to buffer some of the damage and suppress excessive inflammatory responses [54,55].

However, the toxicological differences between these two pollutants have not been clearly revealed, and the underlying reason may be related to the fact that neonatal rats are at a critical stage of alveolar development in their lungs [56].

Therefore, no significant differences were observed between the two groups in terms of oxidative stress or inflammatory factor expression, which was likely because the structure and function of the developing lung tissue had not yet fully matured. Once the damage reached a certain threshold, the lung tissue became less sensitive to further increases in the pollutant concentration.

In addition to the findings presented, this study did not thoroughly explore the upstream and downstream mechanisms of inflammatory factors. To comprehensively understand the impacts of nanoscale iron–chlorobenzene mixtures and their photocatalytic products on lung development and inflammatory responses, future research should delve into the regulatory networks of inflammatory factors, including the activation of upstream signaling pathways and the expression of downstream effector molecules. This approach will help to elucidate the specific mechanisms by which these pollutants influence pulmonary inflammation. Additionally, investigating whether short-term exposure to these pollutants directly affects lung function in adult organisms will be a key objective of our future studies.

## 5. Conclusions

In summary, this study provides evidence of the impact of nanoscale iron–chlorobenzene mixtures before and after photocatalysis on the pulmonary health of rat pups, particularly in terms of inducing oxidative stress and reactive oxygen species (ROS) accumulation, as well as the expression of inflammatory factors. These pollutants exacerbate pulmonary injury by promoting ROS accumulation, inhibiting the expression of acute inflammatory factors and impairing lung repair mechanisms. Both the pre- and post-photocatalytic nanoscale iron–chlorobenzene mixtures had significant effects on early lung development. Given these findings, it is crucial to take further action to control the formation of such pollutants and protect vulnerable populations, especially children and adolescents in critical periods of lung development, from environmental pollution.

## Figures and Tables

**Table 1:** Sequences of the primers used for qRT–PCR.

Name	Forward Primer (5′→3′)	Reverse Primer (5′→3′)
β-actin	ACCCGCGAGTACAACCTTCTT	TCGTCATCCATGGCGAACTGG
IL-6	GCAAGAGACTTCCAGCCAGT	TGCCATTGCACAACTCTTTTC

## Data Availability

The original contributions presented in this study are included in the article. Further inquiries can be directed to the corresponding author(s).

## Abbreviations

The following abbreviations are used in this manuscript:

AhR	Aryl hydrocarbon receptor
POPs	Persistent organic pollutants
PCDD/Fs	Polychlorinated dibenzo-p-dioxins and dibenzofurans
PCBs	Polychlorinated biphenyls
CB	Chlorobenzene
TCDD	2,3,7,8-tetrachlorodibenzo-p-dioxin
PPI	Protein–protein interaction
GO	Gene Ontology
KEGG	Kyoto Encyclopedia of Genes and Genomes
BP	Biological process
CC	Cellular component
MF	Molecular function
FDR	False discovery rate
ROS	Reactive oxygen species
SOD	Superoxide dismutase
TNF	Tumor necrosis factor
IL-17	Interleukin-17
TLR	Toll-like receptor
IL-1β	Interleukin-1β
IL-8	Interleukin-8
IL-6	Interleukin-6

## References

[1] Xing Y., Zhang H., Su W., Wang Q., Yu H., Wang J., Li R., Cai C., Ma Z. (2019). The bibliometric analysis and review of dioxin in waste incineration and steel sintering. Environ. Sci..

[2] Zhan M.-X., Xu S., Cai P., Chen T., Lin X., Buekens A., Li X.J.C. (2019). Parameters affecting the formation mechanisms of dioxins in the steel manufacture process. Chemosphere.

[3] Gouin T., Mackay D., Jones K.C., Harner T., Meijer S.N. (2004). Evidence for the “grasshopper” effect and fractionation during long-range atmospheric transport of organic contaminants. Environ. Pollut..

[4] Xu S., Chen T., Buekens A., Li X.J.I.I. (2018). De novo formation of PCDD/F during sintering: Effect of temperature, granule size and oxygen content. ISIJ Int..

[5] Zhang Y., Li C., Huang Q., Liu X., Zhao J., Zhang Z., Zhu Y., Huang L., Yang K. (2025). Enhancing synergistic catalytic combustion for the co-removal of PCDD/Fs and additional pollutants from sintering flue gases: A review on catalyst development. Sep. Purif. Technol..

[6] Li C., Liu G., Qin S., Zhu T., Song J., Xu W. (2023). Emission reduction of PCDD/Fs by flue gas recirculation and activated carbon in the iron ore sintering. Environ. Pollut..

[7] Ren Z., Lu Y., Li Q., Sun Y., Wu C., Ding Q. (2018). Occurrence and characteristics of PCDD/Fs formed from Chlorobenzenes production in China. Chemosphere.

[8] Baker J.I., Hites R.A. (2000). Is combustion the major source of polychlorinated dibenzo-p-dioxins and dibenzofurans to the environment? A mass balance investigation. Environ. Sci. Technol..

[9] Kulkarni P.S., Crespo J.G., Afonso C.A. (2008). Dioxins sources and current remediation technologies—A review. Environ. Int..

[10] Rogula-Kozłowska W. (2016). Size-segregated urban particulate matter: Mass closure, chemical composition, and primary and secondary matter content. Air Qual. Atmos. Health.

[11] Kosco J., Bidwell M., Cha H., Martin T., Howells C.T., Sachs M., Anjum D.H., Gonzalez Lopez S., Zou L., Wadsworth A.J.N.M. (2020). Enhanced photocatalytic hydrogen evolution from organic semiconductor heterojunction nanoparticles. Nat. Mater..

[12] Wang Q., Liu S. (2023). The effects and pathogenesis of PM_2.5_ and its components on chronic obstructive pulmonary disease. Int. J. Chronic Obstr. Pulm. Dis..

[13] Kajekar R. (2007). Environmental factors and developmental outcomes in the lung. Pharmacol. Ther..

[14] Rice M.B., Rifas-Shiman S.L., Litonjua A.A., Oken E., Gillman M.W., Kloog I., Luttmann-Gibson H., Zanobetti A., Coull B.A., Schwartz J. (2016). Lifetime Exposure to Ambient Pollution and Lung Function in Children. Am. J. Respir. Crit. Care Med..

[15] Pinkerton K.E., Joad J.P. (2000). The mammalian respiratory system and critical windows of exposure for children’s health. Environ. Health Perspect..

[16] Selevan S.G., Kimmel C.A., Mendola P. (2000). Identifying critical windows of exposure for children’s health. Environ. Health Perspect..

[17] Benjamin J.T., Plosa E.J., Sucre J.M., van der Meer R., Dave S., Gutor S., Nichols D.S., Gulleman P.M., Jetter C.S., Han W. (2021). Neutrophilic inflammation during lung development disrupts elastin assembly and predisposes adult mice to COPD. J. Clin. Investig..

[18] Newbury J.B., Arseneault L., Beevers S., Kitwiroon N., Roberts S., Pariante C.M., Kelly F.J., Fisher H.L. (2019). Association of air pollution exposure with psychotic experiences during adolescence. JAMA Psychiatry.

[19] Sangkham S., Phairuang W., Sherchan S.P., Pansakun N., Munkong N., Sarndhong K., Islam M.A., Sakunkoo P.J.E.A. (2024). An update on adverse health effects from exposure to PM_2.5_. Environ. Adv..

[20] Huang S. (2023). Efficient analysis of toxicity and mechanisms of environmental pollutants with network toxicology and molecular docking strategy: Acetyl tributyl citrate as an example. Sci. Total Environ..

[21] Yoshioka W., Tohyama C. (2019). Mechanisms of developmental toxicity of dioxins and related compounds. Int. J. Mol. Sci..

[22] Mishra M., Chun D.-M. (2015). α-Fe2O3 as a photocatalytic material: A review. Appl. Catal. A Gen..

[23] Liu K., Ying M., Chen M., Yan M., Xu Y., Li Y. (2024). Photochemical conversion enhancement of the damaging effects of iron trioxide/chlorobenzene complexes on A549 cells. J. Environ. Sci..

[24] Hulin M., Sirot V., Vasseur P., Mahe A., Leblanc J.C., Jean J., Marchand P., Venisseau A., Le Bizec B., Rivière G. (2020). Health risk assessment to dioxins, furans and PCBs in young children: The first French evaluation. Food Chem. Toxicol. Int. J. Publ. Br. Ind. Biol. Res. Assoc..

[25] Wang L., Pei Y., Li S., Zhang S., Yang Y. (2019). Distinct molecular mechanisms analysis of three lung cancer subtypes based on gene expression profiles. J. Comput. Biol..

[26] Zuehlke A.D., Beebe K., Neckers L., Prince T. (2015). Regulation and function of the human HSP90AA1 gene. Gene.

[27] Chang Z., Lu M., Kim S.-S., Park J.-S.J.T.L. (2014). Potential role of HSP90 in mediating the interactions between estrogen receptor (ER) and aryl hydrocarbon receptor (AhR) signaling pathways. Toxicol. Lett..

[28] Zhang W., Xie H.Q., Li Y., Zhou M., Zhou Z., Wang R., Hahn M.E., Zhao B. (2022). The aryl hydrocarbon receptor: A predominant mediator for the toxicity of emerging dioxin-like compounds. J. Hazard. Mater..

[29] Wee P., Wang Z.J.C. (2017). Epidermal growth factor receptor cell proliferation signaling pathways. Cancers.

[30] Niu Z., Zhao Q., Cao H., Yang B., Wang S.J.S.R. (2025). Hypoxia-activated oxidative stress mediates SHP2/PI3K signaling pathway to promote hepatocellular carcinoma growth and metastasis. Sci. Rep..

[31] Wu M., Bian Q., Liu Y., Fernandes A.F., Taylor A., Pereira P., Shang F. (2009). Sustained oxidative stress inhibits NF-κB activation partially via inactivating the proteasome. Free Radic. Biol. Med..

[32] Lotfy M., Khattab A., Shata M., Alhasbani A., Khalaf A., Alsaeedi S., Thaker M., Said H., Toumi H.R., Alzahmi H.J.H. (2024). Melatonin increases AKT and SOD gene and protein expressions in diabetic rats. Heliyon.

[33] Shen M., Guo M., Wang Z., Li Y., Kong D., Shao J., Tan S., Chen A., Zhang F., Zhang Z.J.I.I. (2020). ROS-dependent inhibition of the PI3K/Akt/mTOR signaling is required for Oroxylin A to exert anti-inflammatory activity in liver fibrosis. Int. Immunopharmacol..

[34] Guo Q., Jin Y., Chen X., Ye X., Shen X., Lin M., Zeng C., Zhou T., Zhang J.J.S.T., Therapy T. (2024). NF-κB in biology and targeted therapy: New insights and translational implications. Signal Transduct..

[35] Aluri J., Cooper M.A., Schuettpelz L.G.J.C. (2021). Toll-like receptor signaling in the establishment and function of the immune system. Cells.

[36] Huangfu L., Li R., Huang Y., Wang S.J.S.T., Therapy T. (2023). The IL-17 family in diseases: From bench to bedside. Signal Transduct. Target. Ther..

[37] Matsumura F., FA Vogel C. (2006). Evidence supporting the hypothesis that one of the main functions of the aryl hydrocarbon receptor is mediation of cell stress responses. Biol. Chem..

[38] Tian Y., Ke S., Denison M.S., Rabson A.B., Gallo M.A. (1999). Ah receptor and NF-κB interactions, a potential mechanism for dioxin toxicity. J. Biol. Chem..

[39] Pernomian L., Duarte-Silva M., de Barros Cardoso C.R. (2020). The aryl hydrocarbon receptor (AHR) as a potential target for the control of intestinal inflammation: Insights from an immune and bacteria sensor receptor. Clin. Rev. Allergy Immunol..

[40] Chen M., Yin M., Su Y., Li R., Liu K., Wu Z., Weng X. (2023). Atmospheric heterogeneous reaction of chlorobenzene on mineral α-Fe2O3 particulates: A chamber experiment study. Front. Environ. Sci. Eng..

[41] Karlsson H.L., Cronholm P., Gustafsson J., Möller L. (2008). Copper oxide nanoparticles are highly toxic: A comparison between metal oxide nanoparticles and carbon nanotubes. Chem. Res. Toxicol..

[42] Song X., Gu X., Sun H., Fu C., Zhang Y., Dong P. (2016). Biomimetic Modification and In Vivo Safety Assessment of Superparamagnetic Iron Oxide Nanoparticles. J. Nanosci. Nanotechnol..

[43] Yan Z., Liu Z., Yang B., Zhu X., Song E., Song Y. (2024). Long-term pulmonary iron oxide nanoparticles exposure disrupts hepatic iron-lipid homeostasis and increases plaque vulnerability in ApoE^−/−^ mice. Environ. Pollut..

[44] Khalil I., Yehye W.A., Etxeberria A.E., Alhadi A.A., Dezfooli S.M., Julkapli N.B.M., Basirun W.J., Seyfoddin A.J.A. (2019). Nanoantioxidants: Recent trends in antioxidant delivery applications. Antioxidants.

[45] Kozlov A.V., Javadov S., Sommer N.J.A. (2024). Cellular ROS and Antioxidants: Physiological and Pathological Role. Antioxidants.

[46] Chelombitko M.A. (2018). Role of reactive oxygen species in inflammation: A minireview. Mosc. Univ. Biol. Sci. Bull..

[47] Niu B., Liao K., Zhou Y., Wen T., Quan G., Pan X., Wu C.J.B. (2021). Application of glutathione depletion in cancer therapy: Enhanced ROS-based therapy, ferroptosis, and chemotherapy. Biomaterials.

[48] Jomova K., Raptova R., Alomar S.Y., Alwasel S.H., Nepovimova E., Kuca K., Valko M. (2023). Reactive oxygen species, toxicity, oxidative stress, and antioxidants: Chronic diseases and aging. Arch. Toxicol..

[49] Profumo E., Buttari B., Tinaburri L., D’Arcangelo D., Sorice M., Capozzi A., Garofalo T., Facchiano A., Businaro R., Kumar P. (2018). Oxidative Stress Induces HSP90 Upregulation on the Surface of Primary Human Endothelial Cells: Role of the Antioxidant 7,8-Dihydroxy-4-methylcoumarin in Preventing HSP90 Exposure to the Immune System. Oxidative Med. Cell. Longev..

[50] Chen Y., Liu C., Tong J., He C., Ling X., Xiang J., Xue C., Yao G., Sun L., Xie Z. (2025). Inhibition of Cullin3 Neddylation Alleviates Diabetic Retinopathy by Activating Nrf2 Signaling to Combat ROS-Induced Oxidative Stress and Inflammation. Inflammation.

[51] Chepurnova D., Samoilova E., Anisimov A., Verin A., Korotaeva A.A. (2018). Compounds of IL-6 receptor complex during acute lung injury. Bull. Exp. Biol. Med..

[52] Liu Y., Chen C.C. (2024). Impact of interleukin 6 levels on acute lung injury risk and disease severity in critically ill sepsis patients. World J. Clin. Cases.

[53] Li C.L., Qi G.H., Peng P., Fang R.J. (2018). Research progress of aryl hydrocarbon receptor in regulating intestinal inflammation. Chin. J. Anim. Nutr..

[54] Lopes F., Coelho F.M., Costa V.V., Vieira É.L., Sousa L.P., Silva T.A., Vieira L.Q., Teixeira M.M., Pinho V. (2011). Resolution of neutrophilic inflammation by H_2_O_2_ in antigen-induced arthritis. Arthritis Rheumatism.

[55] Miller M.D., Marty M.A. (2010). Impact of environmental chemicals on lung development. Environ. Health Perspect..

[56] Negretti N.M., Plosa E.J., Benjamin J.T., Schuler B.A., Habermann A.C., Jetter C.S., Gulleman P., Bunn C., Hackett A.N., Ransom M. (2021). A single-cell atlas of mouse lung development. Development.

